# Cerebral air embolism in interventional pulmonology

**DOI:** 10.3389/fmed.2026.1796947

**Published:** 2026-03-23

**Authors:** Qin Cao, Feng-Lian Song, Dan Liu, Qin-Xue Hu, Duo Li

**Affiliations:** 1 Department of Pulmonary and Critical Care Medicine, The Affiliated Hospital of Southwest Medical University, Luzhou, Sichua, China; 2 Department of Critical Care Medicine, The Affiliated Hospital of Southwest Medical University, Luzhou, Sichuan, China; 3 Department of Infection Management, The Affiliated Hospital of Southwest Medical University, Luzhou, Sichuan, China

**Keywords:** cerebral air embolism, diagnosis, interventional pulmonology, management, pathogenesis

## Abstract

Interventional pulmonology has completely changed the diagnosis and treatment of respiratory diseases through the application of minimally invasive techniques. Cerebral air embolism (CAE) is a potentially fatal complication in this field, with an incidence of only 0.02 to 0.4%, but a mortality rates up to 20 to 30%. From January 2001 to December 2024, we systematically searched PubMed, Scopus, Web of Science, Wanfang, CNKI and VIP and other databases, supplemented by manual retrieval. We included human case reports related to cerebral air embolism induced by interventional pulmonology. This analysis systematically reviewed 53 cases of cerebral venous and arterial air embolism. This review integrates the latest evidence on the epidemiology, pathophysiology, clinical manifestations, and treatment of CAE, with a specific focus on its relationship to interventional pulmonology. By systematically linking pathophysiological mechanisms to specific procedural steps. We propose an evidence-based, procedure-specific preventive framework. Implementing this framework within the standard workflow of interventional pulmonology is essential for anticipating risks, guiding timely diagnosis and intervention, and ultimately improving patient safety in this rapidly evolving field.

## 1 Introduction

### 1.1 Definition and development of interventional pulmonology

Since Mathur and Beamis first introduced the term “interventional pulmonology” in Clinics in Chest Medicine in 1995, the field has gradually evolved into a systematic discipline (1). In 2002, the European Respiratory Society (ERS) and the American Thoracic Society (ATS) jointly defined it as “a system of minimally invasive diagnostic and therapeutic techniques requiring specialized training,” which marked the official launch of its standardization process.

Driven by continuous technological advances, the category of interventional pulmonology has gradually expanded from an initial focus on airway interventions to a comprehensive framework addressing pathologies of the airways, lung parenchyma and pleural space. In 2020, Wahidi (2) and colleagues systematically built a modern interventional respiratory pathology technical framework. In 2022, the Zuccatosta team further promoted the standardization of relevant operating terms (3). Today, interventional pulmonology has evolved into a respiratory medicine subspecialty emphasizing standardized protocols and minimally invasive approaches. A detailed classification of its procedures is presented in Table 1 (4–8). However, the increasing diversity and complexity of these procedures, particularly those involving transparenchymal access, thermal ablation, and artificial pneumothorax, have brought renewed focus to rare but catastrophic complications. Among these, cerebral air embolism (CAE) stands out as a critical safety challenge, demanding a procedure-specific understanding of its risk factors and prevention.

**Table 1:** Classification of procedures in interventional pulmonology.

**Intervention path**	**Diagnosis/stage**	**Treatment**
Bronchoscopy	Bronchoscopic biopsy (biopsy forceps, brush biopsy, lavage, needle aspiration) BAL (Bronchoalveolar Lavage) EBUS-TBNA (Endobronchial Ultrasound-guided Transbronchial Needle Aspiration) ENB (Electromagnetic Navigation Bronchoscopy) Bronchoscopic Cryobiopsy Imaging-assisted techniques:VBN (Virtual Navigation), Narrow-band/fluorescence/confocal microscopy, OCT (Optical Coherence Tomography)	Rigid Bronchoscopy Mechanical Clearance, Bronchoscopic Ablation, Balloon Dilation Bronchoplasty Airway Stent Placement^*^
Percutaneous puncture	CT-guided TTNA (CT-guided Trans-Thoracic Needle Aspiration) Percutaneous Pleural Biopsy	Percutaneous Ablation^*^ Percutaneous Radioactive Particle Implantation Percutaneous Tracheostomy Dilation Therapeutic Thoracentesis
Thoracoscopic	Pleural Cavity Lavage Thoracoscopic Examination VATS (Video-Assisted Thoracoscopic Surgery, Diagnosis and Staging)	Thoracoscopic Drainage Pleurodesis (Talc/Medications) Medical Thoracoscopy (Adhesion Release/Tumor Resection) VATS Treatment (Lung Wedge Resection/Lymph Node Dissection)
Endovascular	Pulmonary angiography	BAI (Bronchial Artery Infusion) BACE (Bronchial Artery Chemoembolization)
Other access routes	Direct/Indirect Laryngoscopy EUS Biopsy (Endoscopic Ultrasound Biopsy for Mediastinal Lesion Evaluation)	/

^*^Ablation therapy includes thermal ablation (radiofrequency, microwave, high-intensity focused ultrasound, laser ablation), cryoablation, brachytherapy, chemoablation, photodynamic therapy, etc.

### 1.2 Definition of cerebral air embolism

With the wide application of interventional respiratory technology and the increasing complexity of operations, the clinical understanding of their associated complications has concurrently deepened. As one of the rare but fatal complications, cerebral air embolism has attracted more and more attention. It is defined as an acute vascular obstruction caused by air entering the cerebral circulatory system. Although its incidence remains low, cerebral air embolism can precipitate acute cerebral ischemia, neurological function defects and even death, and establishing it as one of the most serious complications in interventional pulmonary procedures.

## 2 Epidemiology

Procedure-related complications in interventional pulmonology most commonly include hemorrhage, with an incidence of 5%−15%, and pneumothorax, occurring in 2%−10% of cases. In contrast, the incidence of cerebral air embolism is significantly lower, reported to be between 0.02 and 0.4% (9–12), but its high mortality rate warrants considerable clinical attention. There are differences in the incidence of disease reported in different literatures, the possible reasons likely reflect differences in procedural characteristics, patient-specific factors, and diagnostic confirmation methods. This review includes 53 cases of cerebral air embolism, with a predominance of male patients (male-to-female ratio of 2.1:1) and a mean age of 66.2 years. Among them, Computed tomography-guided percutaneous needle biopsy was the most frequently associated procedure (60.4%), followed by transbronchial lung biopsy (20.8%) and artificial pneumothorax induction for medical thoracoscopy (13.2%) (Table 2).

**Table 2:** Summary of clinical characteristics and outcomes in 53 cases of cerebral air embolism.

**Category**	**Characteristics**	**Value (*n* = 53)**
Demographics	Male: Female (Ratio)	31: 15 (2.1:1)
	Mean age, years (Range)	66.2 (25–88)
Interventional procedure	CT-guided Percutaneous Needle Biopsy (CT-PNB)	32 (60.4%)
	Transbronchial Lung Biopsy/Needle Aspiration (TBLB/TBNA)	11 (20.8%)
	Artificial Pneumothorax for Medical Thoracoscopy	7 (13.2%)
	Bronchoscopic Argon Plasma Coagulation (APC)/Thermal ablation	3 (5.7%)
	Others (Pleural Lavage, IPC exchange, etc.)	3 (5.7%)
Clinical manifestations	Altered Consciousness (Coma, Confusion)	37 (69.8%)
	Focal Neurological Deficits (Hemiplegia, Aphasia)	34 (64.2%)
	Seizures	23 (43.4%)
	Cardiovascular Symptoms (Hypotension, Arrhythmia)	25 (47.2%)
	Respiratory Symptoms (Dyspnea, Hypoxia)	15 (28.3%)
Primary treatment	Hyperbaric Oxygen Therapy (HBOT)	28 (52.8%)
	High-Flow Oxygen Therapy & Fluid Resuscitation	25 (47.2%)
	Mechanical Ventilation/Advanced Life Support	11 (20.8%)
	No Specific/Conservative Treatment	5 (9.4%)
Final outcome	Recovered/resolved	28 (52.8%)
	Neurological Sequelae (Hemiparesis, Cognitive Deficit)	15 (28.3%)
	Died	10 (18.9%)

Data are presented as number (percentage) unless otherwise specified. Percentages may not sum to 100%.

Advances in interventional technology have led to an increase in clinical reports of cerebral embolism, but accurate estimation of its incidence remains challenging. This uncertainty stems primarily from the underdiagnosis of non-specific presentations, such as transient dizziness or visual disturbances, and insufficient postoperative neuroimaging surveillance. Additionally, some cases can only be definitively diagnosed by postmortem examination. Therefore, the actual incidence is likely to be higher than current epidemiological data suggest (13–15), especially in settings without routine neuroimaging protocols.

## 3 Pathogenesis

**Figure 1:**
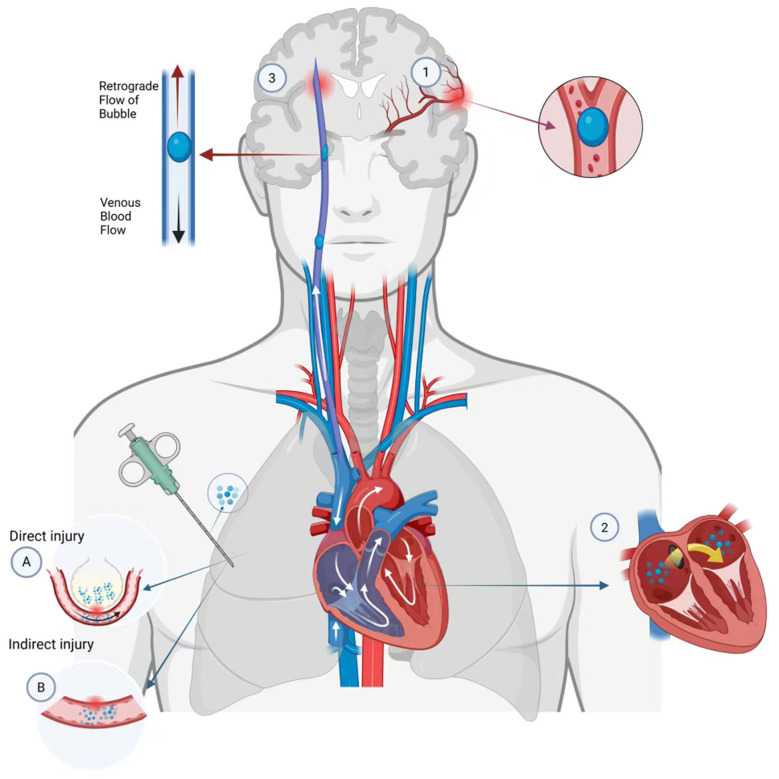
Pathogenic mechanisms of cerebral air embolism in interventional pulmonology. Cerebral air embolism originates from direct or indirect vascular injury during procedures. **(A)** Direct injury involves needle penetration of pulmonary or bronchial veins, allowing immediate gas entry. **(B)** Indirect injury results from alveolar-venous fistula formation, where elevated alveolar pressure during coughing or ventilation drives air into adjacent veins. Subsequent embolization may occur via three principal routes: (1) Direct arterial pathway—gas introduced into a pulmonary vein travels via the left heart to the cerebral arteries; (2) Venous-to-arterial (paradoxical) pathway—venous gas exceeding the pulmonary capillary filtration threshold may cross the pulmonary vasculature or, in the presence of a right-to-left shunt (e.g., patent foramen ovale), bypass it to reach the systemic circulation; (3) Retrograde venous pathway—with rapid gas infusion into cervical veins in an upright patient, buoyant bubbles ascend against flow through the internal jugular vein into the cerebral venous system. (Schematic integrates pathophysiological concepts from references 3, 18–22, 26.) (Created with BioRender.com).

The occurrence of cerebral air embolism primarily results from the introduction of gas into the systemic arterial circulation through abnormal pathways, and eventually reaching the cerebral blood vessels. Gas can enter the cerebral blood vessels through three principal routes:

The direct arterial pathway involves the immediate introduction of gas into the pulmonary artery during an interventional procedure, or it enters the pulmonary vein through the iatrogenic alveolar-venous fistula. The gas subsequently travels via the left heart into the aorta, leading to direct cerebral arterial embolism (16). The venous-to-arterial pathway, often termed paradoxical embolism, refers to the gas entering the venous system first. If the volume is sufficient enough (exceeding about 0.40 ml/kg/min, the filtration threshold of pulmonary capillaries), it may pass through the pulmonary vascular system into the left ventricle (17). Additionally, for patients with right-to-left shunt such as a patent foramen oval (PFO), when the right atrial pressure rises, the venous gas can enter the systemic circulation directly, resulting in paradoxical embolism (18). The retrograde venous pathway is a special mechanism, in which a large volume of gas rapidly infuses into cervical veins while the patient is in an upright position. Buoyancy bubbles may reverse venous blood flow into the intracranial venous system through the internal jugular vein, causing cerebral venous embolism (19). Regardless of the entry path, the presence of gas in the cerebral circulation will trigger the key pathological sequence at the gas-blood interface and trigger the complement-mediated thromboinflammatory reaction. The three principal routes of gas entry into the cerebral circulation are illustrated in Figure 1. This process induces endothelial damage, platelet activation, amplification of the coagulation cascade and cytokine storm, which eventually leads to cerebral ischemia injury. The specific damage mechanism can be summarized as follows:

### 3.1 Vascular injury and gas invasion

Direct vascular injury is the primary route of gas invasion. When air-containing needles or instruments penetrate the pulmonary or bronchial veins, they will form a direct communication, permitting air to enter the circulatory system (11). Indirect injury usually originates from the formation of alveolar-venous fistula, that is, a single puncture passes through the alveoli and adjacent pulmonary veins at the same time. During coughing or positive pressure ventilation, increased alveolar pressure may drive gas into the venous system through the fistula (11, 20).

### 3.2 Hemodynamic abnormalities

Hemodynamic changes significantly influence the gas entrainment risk. In states of hypovolemia, hypotension (21) or shock, a decrease in central venous pressure will amplify the transmural pressure difference and promote air entry into the venous system (22). If the rate of gas entry exceeds the pulmonary capillary filtration capacity, it may cause systemic embolism (11). In such cases, cardiac function also plays a key role: the enhancement of right ventricular contractility can accelerate the transfer of gas from the right heart to the left heart, significantly increasing the risk of systemic embolism, especially in patients with patent foramen oval (18, 23).

### 3.3 Procedure-related risks

Technical factors in the intervention operation are important triggers. Different interventional procedures have different risk characteristics due to their specific mechanisms of vascular injury and gas introduction.

#### 3.3.1 CT-guided transthoracic needle aspiration (TTNA)

The direct arterial pathway is most pertinent to the risk of TTNA. As described above, the withdrawal of the stylet or coaxial needle under a non-sealed system can create a direct communication between atmospheric air and a punctured pulmonary vein. If the needle passes through the alveoli and vein at the same time, an alveolar-venous fistula will be formed. Against this background, a patient's cough or a sudden increase in airway pressure during the procedure can acutely increase alveolar pressure, driving air directly into the low-pressure pulmonary venous system, this mechanism is frequently implicated in post-biopsy CAE (11, 20). Failure to promptly occlude the puncture channel upon needle removal further aggravate this risk (24).

#### 3.3.2 Bronchoscopic thermal ablation (APC/Laser)

Procedures utilizing thermal energy, such as argon plasma coagulation (APC) or laser ablation, introduce unique risks. High-energy or long-term pulse settings can cause direct tissue necrosis invading the adjacent pulmonary vein wall, creating a portal for gas entry (25, 26). In these cases, the insufflated gas itself can be a direct source of embolism and enter the circulatory system through newly formed vascular defects. This mechanism involves both direct vascular injury and improper pressure management, as the insufflation pressure may exceed the venous pressure, promoting gas invasion (24).

#### 3.3.3 Medical thoracoscopy with artificial pneumothorax

During medical thoracoscopy, the induction of an artificial pneumothorax presents a specific situation for the retrograde venous pathway. If gas is insufflated too rapidly or at excessive pressure into the pleural space, and if a concurrent injury to a thoracic vein exists, the gas may be forced retrograde into the cerebral venous system. When the patient takes a semi-upright position, the risk increases significantly, as he buoyancy action to promote retrograde rise (19, 27). Additionally, if the patient has a history of pleural adhesion, localized high-pressure zones may form, which further increases the probability of gas spreading retrogradely to the vascular structure.

### 3.4 Patient-specific risk factors

Individual anatomical and physiological characteristics significantly affect the susceptibility of embolism. Anatomical malformations, including right-to-left shunt (patent foramen oval, ventricular septal defect) and pulmonary arteriovenous malformations (27–29), will reduce pulmonary filtration efficiency (30) and promote gas into the body circulation (24). Cavity lesions such as pulmonary cysts increase the air–blood interface area and increase invasion potential. Concurrent coagulation abnormalities, such as a platelet count < 50 × 10^9^/L or INR >1.5, will delay the physiological closure of vascular defects, prolonging the window for gas entry and increasing embolization risk.

## 4 Clinical manifestations

In the context of interventional pulmonology (IP), the occurrence of cerebral air embolism (CAE) is often dramatic and closely synchronized with specific procedural maneuvers. Our analysis of 53 cases showed that 69.8% of patients had acute consciousness disturbances immediately following needle withdrawal or high-pressure gas insufflation. The typical feature of IP-related CAE is the Cough, a sudden, forceful cough during the procedure, which acutely elevates intrathoracic pressure and drives air into the pulmonary venous system, followed by an immediate loss of consciousness and subsequent tonic-colonic seizures. The severity of symptoms is closely related to the volume of embolized gas, the affected anatomical area and the basic physiological state of the patient. In addition to the specific symptoms of the interventional pulmonology (IP), the clinical manifestations of CAE cover a wide spectrum, from subtle non-specific signs to life-threatening acute cardiovascular failure. According to the main affected organ systems, these manifestations can be classified into several areas.

### 4.1 Neurological symptoms

Consciousness impairment is frequently observed, often manifested as sudden coma or altered mental status, which is commonly related to frontal lobe or brainstem embolism. Some patients have immediate unresponsiveness during surgery or shortly after surgery (31–34). Focal neurological deficits (such as hemiplegia, aphasia, or visual field defects) are also common, and some cases have unilateral limb weakness or numbness after embolism (33, 35–38). Additionally, seizures may occur, ranging from generalized tonic-clonic to focal seizures, and may progress to status epilepticus in severe cases (31, 32, 39–42).

### 4.2 Cardiovascular symptoms

When air embolism affects the coronary circulation or compromises the systemic hemodynamics, various arrhythmias may occur, including sinus tachycardia and premature ventricular contractions. Severe cases may progress to sustained ventricular arrhythmias or cardiac arrest (26, 32, 43–45). Some patients have signs of myocardial ischemia, such as chest pain or changes in the ST-segment of the electrocardiogram (44), while others have hemodynamic instability, including hypotension or obvious shock (25, 43, 46–48).

### 4.3 Respiratory symptoms

Air entering the pulmonary circulation may cause acute dyspnea and hypoxemia (SpO2 < 90%), and some patients develop cyanosis or tachypnea during surgery (25, 37, 49, 50). Cough or hemoptysis may also occur in a minority of cases, suggesting gas entry into the bloodstream through a pulmonary-venous fistula (31, 51).

### 4.4 Atypical manifestations

Some patients present with nonspecific systemic symptoms, such as nausea, vomiting, or abdominal pain, which may reflect the systemic stress response to the embolism (52). It is worth noting that not all cases show typical neurological signs; for example, the non-fatal CAE cases reported by Hiraki et al. (11) lacked overt neurological dysfunction, highlighting the condition's variable clinical presentation and underscoring the importance of maintaining a high index of suspicion even in the absence of classic symptoms.

## 5 Imaging features

Imaging examination plays a key role in the diagnosis and evaluation of cerebral air embolism. Cranial computed tomography (CT) and magnetic resonance imaging (MRI) are the core examination methods. Typical imaging manifestations include multiple, bilateral, irregular, and asymmetrical lesions, often involving the cortical region of the frontal lobe and parietal lobe (25, 50).

### 5.1 Cranial CT (non-contrast scan)

Non-contrast cranial CT is the preferred method for rapid screening. The detection of low-density gas collections in the brain parenchyma, termed “bubble sign” (53), can serve as a direct diagnostic indicator. However, the early sensitivity of CT is still relatively low (about 40%). Therefore, for clinical suspicious cases, if the initial scan result is negative, it is recommended to review the imaging examination within 6 h after the onset of symptoms to improve the detection rates.

### 5.2 MRI diffusion-weighted imaging (DWI)

Diffusion-weighted imaging has high sensitivity for detecting early and subtle ischemic lesions (54). In most cases, characteristic punctate or patchy areas of restricted diffusion become visible on DWI sequences within 6–24 h after symptom onset. DWI provides an important basis for defining the scope of infarction and evaluating the severity of tissue injury (55).

### 5.3 Transcranial doppler ultrasound (TCD)

TCD currently stands as the only technique capable of real-time monitoring of circulating microemboli, including both gaseous and solid types. This technology is extensively used to detect arterial microemboli during extracorporeal circulation surgery (56). The characteristic “snowstorm-like” high-intensity signal was observed to strongly indicate air embolism (57). However, due to equipment requirements and operator dependence, the application of TCD in postoperative monitoring of respiratory intervention has not been fully utilized in clinical settings, especially in local practice.

## 6 Differential diagnosis

Cerebral air embolism presents with acute onset and diverse clinical manifestations, and needs to be differentiated from a range of other acute neurological, cardiovascular, metabolic, toxic, and psychogenic disorders.

### 6.1 Neurological diseases

Acute Ischemic Stroke: Typically occurs in patients with cerebrovascular risk factors such as hypertension and diabetes, and without a recent history of respiratory intervention. Imaging demonstrates infarcts within specific vascular territories, without evidence of intracranial gas.

Intracranial Hemorrhage: often presents with sudden severe headache and vomiting. Non-contrast CT shows hyperdense hematoma, which contrasts with hypodense gas.

Status Epilepticus: most patients have epilepsy history; electroencephalography shows epileptic-like discharge during seizure or intermittent period and neuroimaging lacks signs of gas embolism.

Intracranial Infection: usually accompanied by fever, stiff neck and other systemic symptoms. The analysis of cerebrospinal fluid shows cell proliferation and protein elevation, with no radiographic evidence of air (58).

### 6.2 Cardiovascular diseases

Acute Myocardial Infarction: characterized by compression-like retrosternal pain, often radiating to the left arm or jaw. ECG reveals dynamic ST-T segment changes, and cardiac biomarkers are elevated, in the absence of neurological localizing signs or intracranial air (59).

Pulmonary Embolism: manifests as acute dyspnea, pleuritic chest pain and hypoxemia. D-dimer levels are typically elevated, and pulmonary CT angiography can confirm intravascular thrombosis (60).

### 6.3 Metabolic and toxic diseases

Hypoglycemic Coma: this condition is often accompanied by autonomic symptoms such as diaphoresis and palpitations. A rapid blood glucose testing can confirm hypoglycemia (< 3.9 mmol/L), and the symptoms improve rapidly after intravenous glucose injection (61).

Hepatic Encephalopathy: this condition is Occurs in the chronic liver disease, often with asterixis and personality changes. Serum ammonia is elevated, and focal neurological deficits are absent (62).

Toxic Encephalopathy (e.g., Carbon Monoxide Poisoning): the history of poisoning exposure is essential. Brain MRI may show symmetric T2 hyperintensity in the globus pallidus or white matter (63).

## 7 Treatment

Cerebral air embolism (CAE) is a life-threatening emergency requiring immediate recognition and intervention. A recent study reported an overall mortality rate of 46% among patients with CAE, with nearly half of survivors experiencing varying degrees of neurological sequelae (64). Which highlights the importance of timely and appropriate intervention. The core principles of management include immediate prevention of further gas entry, stabilizing vital signs, and targeted elimination of intravascular gas. The treatment should be individualized according to embolism type, severity, and clinical presentation of embolism. A comprehensive therapeutic strategy is outlined below.

### 7.1 Emergency management

#### 7.1.1 Terminate further gas entry

The gas source should be sealed immediately (e.g., by compressing puncture sites or closing an insufflation valve). For patients who receive positive pressure ventilation, it should be considered to switch to spontaneous breathing to reduce alveolar pressure and minimize the risk of gas invasion through vascular defects.

#### 7.1.2 Postural interventions

Suspected Venous Embolism: the Durant position (left lateral decubitus) combined with Trendelenburg positioning is recommended. This position can retain the gas in the right ventricular outflow tract, limit its entry into the pulmonary circulation, and gain time for definitive treatment (65, 66).

Suspected Arterial Embolism or Neurological Symptoms: it is recommended to use the supine position or the simple Trendelenburg position to minimize the migration of gas to the cerebral arteries due to buoyancy (66). In specific mild cases, reasonable position management can relieve symptoms or even completely eliminate them (67).

#### 7.1.3 High-flow oxygen therapy

Administration of 100% oxygen via a tight-fitting mask establishes a steep nitrogen diffusion gradient, promoting the elimination of gas from bubbles and reducing their volume (68). Concurrently, hyperoxygenation can improve cerebral hypoxia resulting from vascular obstruction.

### 7.2 Specific treatment

#### 7.2.1 Hyperbaric oxygen therapy (HBOT)

HBOT represents the first-line specific treatment for confirmed or highly suspected symptomatic cerebral air embolism. Its therapeutic effect comes from three physical and physiological effects:

Bubble compression via elevated ambient pressure (Boyle's law)

Accelerated nitrogen removal and bubble dissolution

Significantly increased the oxygen delivery to ischemic tissue.

Initiation of HBOT should occur as soon as possible; evidence supports that its efficacy even when started within 48 h of the onset of symptoms (25, 69). Notably, untreated pneumothorax constitutes an absolute contraindication due to the risk of tension pneumothorax under hyperbaric conditions (70).

#### 7.2.2 Circulatory gas evacuation

Central Venous Aspiration: when ultrasound confirms right-heart gas, aspiration via a centrally placed catheter positioned at the mid-atrial level may be attempted (16, 71).

Extracorporeal Membrane Oxygenation (ECMO): Veno-arterial or Veno-venous ECMO provides cardiopulmonary support in cases of refractory shock or collapse and facilitates active removal of circulating gas (72, 73).

### 7.3 Systemic supportive care

#### 7.3.1 Hemodynamic support

Crystalloid fluid resuscitation should be actively used to enhance the preload and central venous pressure. It is necessary to titrate vasopressor (such as norepinephrine) to maintain the average arterial pressure ≥65 mmHg to ensure sufficient cerebral perfusion.

#### 7.3.2 Respiratory support

If the patient has respiratory failure or refractory hypoxemia despite high-flow oxygen, it should be upgraded to non-invasive or invasive mechanical ventilation.

#### 7.3.3 Neurological support & protection

Intracranial Pressure Management: in the case of cerebral edema, osmotic agents (such as mannitol and hyperosmotic saline) can be used.

Targeted Temperature Management: therapeutic mild hypothermia (32–36 °C) can be considered for coma patients to attenuate cerebral metabolic needs and inflammatory injury (74, 75).

Seizure Control: Antiseizure medications, such as levetiracetam, should be administered promptly if seizures occur.

#### 7.3.4 Metabolic homeostasis

It is necessary to closely monitor arterial blood gas and electrolytes, and carefully maintain the balance of fluids, electrolytes, and acid-base.

#### 7.3.5 Rehabilitation and long-term prognosis

The recovery of neurological function is often protracted. After the condition is stable, early multimodal rehabilitation treatment should be started within 24–72 h, including physical therapy, occupational therapy, and cognitive therapy. Long-term follow-up is essential and needs to be combined with standardized functional assessment (such as the improved Rankin scale) and a series of neuroimaging examinations (such as MRI) to evaluate recovery and prognosis.

## 8 Prevention

Although cerebral air embolism is rare, it is still a potentially fatal complication of interventional pulmonary procedures. Despite various treatment strategies, CAE was associated with an overall mortality rate of 18.9%. In addition, 28.3% of survivors had permanent neurological sequelae, resulting in a complete recovery rate of only 52.8%. Based on the pathophysiological mechanisms, we have developed a structured prevention framework that integrates preoperative risk anticipation, intraoperative technical optimization, and postoperative surveillance. The framework aims to help clinicians predict, prevent, and rapidly respond to CAE in the interventional pulmonology setting.

### 8.1 Pre-procedural risk anticipation

#### 8.1.1 Patient screening

Right-to-Left Shunt Evaluation: For high-risk individuals (e.g., those with unexplained stroke history or migraine), it is recommended to use bubble contrast echocardiography to detect unclosed foramen (PFO) or pulmonary arteriovenous malformation (9, 76). This move can proactively identify patients at high risk for paradoxical embolism, enabling enhanced intraoperative precautions.

Coagulation Status: It is recommended to perform a comprehensive coagulation test (platelet count >60 × 10^9^/L; INR < 1.5) to ensure physiological closure of vascular defects post-puncture.

#### 8.1.2 Lesion characterization

High-Resolution CT Review: carefully evaluate images to identify cavitary lesions, pulmonary bullae or lesions adjacent to major pulmonary vessels. These findings predict an increased risk of alveolar-venous fistula formation, which should be avoided when planning puncture paths (47, 48, 69).

Airway Assessment: for bronchoscopic interventions, evaluate endobronchial lesions for proximity to vascular structures.

#### 8.1.3 Patient preparation

Patient Education: Patients must be instructed to cooperate with breathing instructions. Patients should be instructed to avoid coughing, deep inspiration, or Valsalva movements during the procedure to maintain stable intrathoracic pressure (11, 69).

Positioning Strategy: the supine position is preferred for percutaneous biopsy to reduce the risk of gas entering the cerebral venous system. Raising the head 15 °-30 ° during bronchoscopy helps to reduce the possibility of gas entering the circulation.

### 8.2 Intra-procedural risk minimization

#### 8.2.1 CT-guided transthoracic needle aspiration (TTNA)

For CT-guided transthoracic needle aspiration (TTNA), maintaining a sealed circuit is crucial; therefore, operators should always use a coaxial biopsy system equipped with a hemostatic valve to prevent atmospheric air from entering during needle manipulation. Upon needle withdrawal, the puncture tract must be immediately occluded with gelatin sponge placement or patient repositioning to minimize the risk of air infiltration (9, 11). Puncture attempts should be limited to ≤ 3 per lesion to reduce the extent of pleural and vascular injury, and the needle trajectory should be carefully planned to avoid traversing pulmonary fissures, bullae, or lesions adjacent to major vessels whenever possible. During the whole procedure, real-time imaging guidance, such as CT fluoroscopy or ultrasound, should be employed to continuously visualize the needle tip and ensure that the vascular structure is avoided.

#### 8.2.2 Bronchoscopic thermal ablation (APC/laser)

In bronchoscopic thermal ablation procedures, including argon plasma coagulation (APC) and laser therapy, the selection of insufflation gas is critical; whenever feasible, CO_2_ should be used instead of air due to its higher blood solubility and consequently lower embolic potential. It is necessary to use low-energy pulse settings to optimize energy transfer to limit the depth of tissue necrosis and reduce the risk of formation of direct vascular portal (25, 26). In order to prevent barotrauma and forced gas entry into injured vessels, the pressure and duration of gas insufflation should be minimized, and particular caution is warranted during visible bleeding, as over-insufflation may drive gas into open veins to cause embolism.

#### 8.2.3 Medical thoracoscopy with artificial pneumothorax

For medical thoracoscopy with artificial pneumothorax induction, a slow and controllable operation method is essential. Gas should be insufflated at a rate of ≤ 500 ml/min to prevent rapid pressure surges that could promote gas dissection into vascular structures (77, 78). During the operation, intrapleural pressure must be continuously monitored and maintained at ≤ 15 cm H_2_O to avoid barotrauma and reduce the risk of gas entering the circulation. Before gas injection, needle position should be verified by aspirating fluid rather than blood. The total volume of gas insufflated should not exceed the volume of preoperative drainage fluid, excessive volume may cause dangerous pressure elevation (77, 78). For patients with minimal pleural fluid or suspected adhesions, clinicians should consider not inducing pneumothorax for operation, if necessary, it must be operated with extreme caution under continuous monitoring (79).

#### 8.2.4 General intraoperative precautions

Ventilation management: in ventilated patients, a lung-protective strategy (tidal volume 6–8 ml/kg, plateau pressure < 30 cm H_2_O) should be applied (75).

Physiologic Monitoring: when the blood oxygen saturation (SpO2) drops to >5%, the operation should be stopped immediately and evaluate the possibility of air embolism—this is an early warning sign of gas entry. Clinicians need to be alert to unexplained hypotension, new arrhythmias, or abrupt changes in end-tidal CO_2_, which may suggest hemodynamic compromise caused by embolism. maintaining hemodynamic stability by avoiding hypovolemia is essential, because reduced central venous pressure amplifies the transmural pressure gradient and promote air to enter the venous system (21, 22).

Real-Time Monitoring: continuous ultrasound or CT perspective guidance can visually display the needle track and avoid the vascular structure.

### 8.3 Post-Procedural surveillance

#### 8.3.1 Immediate post-procedure care

The puncture site should be pressured for ≥30 min. Patients should remain supine for at least 2 h, avoiding coughing or exertion.

#### 8.3.2 Neurologic vigilance

High-risk patients require longer monitoring, and the nervous system is evaluated every 30 min for 6 h. Bedside transcranial Doppler may detect microembolic signals. Any neurological symptoms require urgent head CT examination. Asymptomatic high-risk patients can undergo screening CT within 2 h post-procedure.

#### 8.3.3 Imaging surveillance

Symptomatic patients: immediate head CT to detect intracerebral air (“bubble sign”) (53). If initial CT is negative but still doubtful, we need to review the image within 6 h or proceed to MRI-DWI.

Asymptomatic high-risk patients: consider screening head CT within 2 h post-procedure to detect subclinical gas entry.

### 8.4 Summary: integrating prevention into clinical workflow

The cornerstone of CAE in interventional pulmonology (IP) prevention remains strict adherence to procedural guidelines and contraindications. By anticipating risk through preoperative screening, minimizing risk through intraoperative technical optimization, and detecting risk through postoperative surveillance, clinicians can substantially reduce the incidence of this devastating complication. The professional skills of the operator and the systematic implementation of this standardized workflow are fundamental guarantee to ensure patient safety in interventional pulmonology.

## 9 Limitations

It must be admitted that the current evidence base on cerebral air embolism in the field of interventional pulmonology mainly relies on case reports and case series studies. This introduces an inherent publication bias; wherein catastrophic or fatal outcomes are disproportionately represented in the literature. Therefore, the reported mortality rates (18.9% in our series) and clinical severity may not fully reflect the true spectrum of CAE. In particular, the incidence of mild, transient, or even asymptomatic cases, cases that heal without specific intervention or recognition, is likely underestimated. The lack of routine post-procedural neuroimaging examination for asymptomatic patients further aggravates this certainty bias. Therefore, although our review provides a comprehensive synthesis of reported cases, it still needs to be interpreted carefully when extrapolating to t a wider group of patients with interventional pulmonary procedures. In the future, In the future, it is urgent to carry out prospective research using standardized neurologic monitoring and imaging protocols to establish the true incidence and full clinical spectrum of CAE in this setting.

## 10 Summary and outlook

In summary, cerebral air embolism is a rare but life-threatening complication during pulmonology interventional procedures. Continuous attention to potential risk factors, combined with early recognition and timely intervention, remains crucial to reducing related disability and mortality rates. While the general prevention principle applies, the unique challenges posed by the diversity and complexity of modern interventional pulmonology require a more targeted approach. This review provides a comprehensive, evidence-based framework specifically designed for the interventional pulmonology setting. The systematic implementation of such targeted preventive strategies can significantly reduce the incidence of serious sequelae and fatal outcomes.
