# Supplementary material for: Cerebral air embolism in interventional pulmonology
Source: Front Med (Lausanne). 2026 Mar 23;13:1796947. doi: 10.3389/fmed.2026.1796947 (PMC13050928; doi:10.3389/fmed.2026.1796947)
Supplement: Supplementary file 1 [file Data_Sheet_1.docx]

Table S1. Clinical Characteristics of Patients with Cerebral Air Embolism Complicated by Respiratory Interventional Procedures

| Reporting Year | Authors | Gender | Age | Presentation | Diagnosis | Interventional Procedure | Clinical Manifestations (Cerebral Air Embolism) | Imaging Findings | Treatment | Outcome |
| --- | --- | --- | --- | --- | --- | --- | --- | --- | --- | --- |
| 2007^[1]^ | WANG Lingzhi et al. | Male | 29 | Cough | NA | CT-PNB | Apathy; limb rigidity | Brain CT: Hypodensity in right temporal lobe | High-flow oxygen therapy | Recovered |
| 2008^[2]^ | KE Caixia et al. | NA | NA | NA | NA | Artificial pneumothorax for MT | Consciousness disturbance; tachypnea; opisthotonos | NA | Gas aspiration; Fluid resuscitation; HBOT | Recovered |
| 2009^[3]^ | YU Xiaowei et al. | NA | NA | NA | Pleural effusion (unknown etiology) | Artificial pneumothorax for MT | Altered mental status; cyanosis; facial palsy; left-sided hemiplegia | NA | Gas aspiration; Oxygen therapy; Neurotropic therapy | Recovered |
| 2014^[4]^ | WANG Fang et al. | Male | 85 | Chest discomfort; Dyspnea on exertion | Fibrous tissue hyperplasia | CT-PNB | Sudden unconsciousness (post coughing); irregular breathing; limb convulsions; GCS 6 | Brain CT: Gas densities at gray-white matter junction | Endotracheal intubation; Mechanical ventilation; Anticonvulsants | Discharged against advice |
| 2015^[5]^ | ZHOU Xiaoyu et al. | NA | NA | NA | Tuberculous pleural effusion | Artificial pneumothorax for MT | Transient right-sided hemiplegia | NA | HBOT; Neurotropic therapy | Recovered |
| 2015 | FEI Junjie et al. | Female | 67 | Empyema (post-esophagectomy) | Esophageal cancer | Pleural Lavage | Right lower limb numbness; weakness (conscious) | NA | No specific treatment (spontaneous resolution within 30 mins) | Recovered |
| 2015 | FEI Junjie et al. | Male | 74 | Thoracic infection (post-esophagectomy) | Esophageal cancer | Pleural Lavage | Sudden confusion; absent light reflex; unresponsiveness | NA | Aspiration of residual pleural fluid/gas; Fluid resuscitation | Recovered |
| 2016^[6]^ | SONG Xianghong et al. | Male | 58 | Pulmonary nodules | Cryptococcus neoformans infection | CT-PNB | Dizziness; left limb paralysis; facial palsy; gaze deviation | Brain MR: Cerebral arterial air embolism | NA | Recovered |
| 2020^[7]^ | WANG Zhaohui et al. | Male | 78 | Cough; Sputum; Dyspnea (worsening) | NA | CT-PNB | Loss of consciousness (2 min) | Brain CT: Multiple gas densities in bilateral frontal, parietal, occipital, temporal sulci | High-flow oxygen therapy; Fluid resuscitation | Recovered |
| 2020^[8]^ | YANG Zhonghua et al. | NA | 71 | NA | NA | TBLB | Coma | Brain CT: Diffuse cerebral air embolism | HBOT | Recovered |
| 1999^[9]^ | Fumiko Kodama et al. | Male | 60 | Pulmonary nodule | NA | CT-PNB | Dyspnea; cyanosis; fatigue; followed by shock | Brain CT: Gas in right ACA and MCA | NA | Died |
| 2001^[10]^ | Satomi Ohashi et al. | Male | 75 | Pulmonary nodule | Lung cancer | CT-PNB | Lethargy; weakness; seizure | Brain CT: Gas bubbles in small vessels of right parietal lobe | HBOT | Neurological sequelae |
| 2001^[11]^ | Prashant G Shetty et al. | Male | 60 | Weight loss | Chronic interstitial pneumonia | TBLB | Status epilepticus | Brain CT: Gas in frontal/parietal convexity regions | HBOT | Died |
| 2004 | Dhillon et al. | Male | 55 | Cough (worsening) | Lung cancer | TBLB | Loss of consciousness; generalized tonic-clonic seizure (1 min); left-sided hemiplegia | Brain CT (30min post): Gas density in right frontal/parietal lobes | High-flow oxygen therapy; Fluid resuscitation | Recovered |
| 2004^[12]^ | G. Ferretti et al. | NA | 62 | Pulmonary nodule | NA | CT-PNB | Dyspnea; loss of consciousness | Chest CT: Gas in left ventricle and aorta; Brain CT: No intracerebral gas; | High-flow oxygen therapy; Fluid resuscitation | Recovered |
| 2006^[13]^ | K. Timpert et al. | Male | 71 | Pulmonary nodule | NA | CT-PNB | Dyspnea; loss of consciousness | Brain CT: Right hemispheric air embolism | High-flow oxygen therapy; Fluid resuscitation | Neurological sequelae |
| 2007^[14]^ | Hiraki et al. | Female | 54 | NA | Pulmonary leiomyoma | CT-PNB | Sudden drop in oxygen saturation | Brain CT: Gas in left frontal lobe | Fluid resuscitation; HBOT | Neurological sequelae |
| 2007^[14]^ | Hiraki et al. | Female | 75 | NA | Pulmonary fibroma | CT-PNB | Restlessness; sudden hypotension | Brain CT: Minimal intravascular gas | Fluid resuscitation; HBOT | Recovered |
| 2007^[14]^ | Hiraki et al. | Male | 71 | NA | Lung cancer | CT-PNB | Right-sided hemiplegia; sudden hypotension | Brain CT: Gas in bilateral frontal and parietal lobes | Fluid resuscitation; HBOT | Recovered |
| 2008^[15]^ | Hirasawa et al. | Female | 69 | Pulmonary nodule | Lung cancer | CT-PNB | Sudden consciousness disturbance; hypotension | Brain CT: Gas in right cerebral arteries | Fluid resuscitation; HBOT | Neurological sequelae |
| 2008^[16]^ | Kau et al. | Male | 50 | Chest shadow | Lung cancer | CT-PNB | Restlessness; diaphoresis; hypotension; transient right-sided hemiplegia | Brain CT: Gas in left frontal and parietal lobes | Fluid resuscitation; HBOT | Recovered |
| 2008^[17]^ | Tomabechi et al. | Male | 71 | Cough; Sputum | Lung cancer | CT-PNB | Restlessness; hypertension (no neurological symptoms) | Chest CT: Gas in left ventricle and aorta; Brain CT: Normal | Fluid resuscitation; HBOT | Recovered |
| 2008^[18]^ | Hsi et al. | Male | 67 | Hip arthroplasty; Recurrent fever; Joint infection | Bronchiolitis obliterans with organizing pneumonia (BOOP) | CT-PNB | Unresponsiveness; limb convulsions | Brain CT: Multiple cerebral air emboli | Fluid resuscitation; HBOT | Recovered |
| 2008^[19]^ | K. Osseiran et al. | Male | 64 | Hepatic fibroma (likely a typo/irrelevant, primary reason unclear) | Lung cancer | APC | Dyspnea; coma | CT: Gas embolism in subdiaphragmatic/sacral region (probable systemic) | Pharmacological management | Died |
| 2009 | Cipriani et al. | Female | 68 | Cough (worsening) | Lung cancer | TBLB | Cardiac arrest; respiratory arrest | Brain CT (Day 2): Gas in subarachnoid space and intracranial vessels | CPR | Died |
| 2009^[20]^ | Um SJ et al. | Male | 40 | Fatigue; Fever; Dyspnea; Weight loss | AIDS-associated Pneumocystis pneumonia | CT-PNB | Dyspnea; coma; generalized tonic-clonic seizure | Brain CT: Gas in bilateral frontal and parietal lobes | Conventional fluid resuscitation | Died |
| 2009^[20]^ | Um SJ et al. | Male | 75 | Chest pain | Sarcomatoid carcinoma | CT-PNB | Sudden consciousness disturbance; limb convulsions; left-sided hemiplegia | Brain CT: Gas in bilateral frontal, parietal, temporal lobes | HBOT; Fluid resuscitation | Died |
| 2009^[20]^ | Um SJ et al. | Female | 67 | Asymptomatic; Pulmonary nodule | Chronic granulomatous inflammation | CT-PNB | Sudden consciousness disturbance; hypotension; left-sided hemiplegia | Brain CT: Gas in left frontal lobe | HBOT; Fluid resuscitation | Recovered |
| 2009^[20]^ | Um SJ et al. | Male | 67 | Cough | Lung cancer | CT-PNB | Restlessness; convulsion; right-sided hemiplegia | Brain CT: Gas in right parietal lobe | HBOT; Fluid resuscitation | Recovered |
| 2010^[21]^ | Azzola et al. | Female | 60 | Hoarseness | Lung cancer | TBLB | Unresponsiveness; GCS 7 | Brain CT: Multiple cerebral air emboli | HBOT (once) then treatment withdrawal | Died |
| 2010^[21]^ | Azzola et al. | Female | 68 | Pulmonary nodule | Lung cancer | TBLB | Sudden consciousness disturbance; anisocoria; hypotension; tachycardia; hypoxia | Brain CT: Extensive air embolism in left hemisphere | Endotracheal intubation; Mechanical ventilation | Died |
| 2010^[22]^ | Bouassaly et al. | NA | 76 | Pulmonary nodule | Lung cancer | CT-PNB | Bradycardia; lethargy | Brain CT: Gas in left frontal, parietal, temporal lobes | HBOT; Fluid resuscitation | Died |
| 2011^[23]^ | Thomas et al. | Female | 25 | Chest pain; Anorexia; Weight loss | Chondroid hamartoma | CT-PNB | Recurrent cardiac arrest | Brain CT: Skull hypodensity; Intravascular gas | Endotracheal intubation; Mechanical ventilation | Died |
| 2011^[24]^ | Shaw et al. | Female | 88 | Lung cancer (known diagnosis) | Lung cancer | APC | Sudden bradycardia; confusion; unresponsiveness | Brain CT: Gas in bilateral frontal and parietal lobes | Endotracheal intubation; Mechanical ventilation | Died |
| 2013^[25]^ | Shi Liuhong et al. | Male | 85 | Weight loss | Pulmonary fibroma | CT-PNB | Unresponsiveness; irregular breathing; hypoxia; limb spasms; GCS 6 | Brain CT: Scattered intraparenchymal gas | Mechanical ventilation; Antispasmodics; Corticosteroids | Discharged against advice |
| 2013^[26]^ | Suzuki et al. | Female | 75 | NA | Lung cancer | CT-PNB | Dizziness; bilateral vision loss | Chest CT: Gas in aorta; Brain CT: Normal | HBOT; Fluid resuscitation | Recovered |
| 2013^[27]^ | Smit et al. | Male | 71 | NA | Lung cancer | CT-PNB | Sudden consciousness disturbance; limb convulsions; bilateral hemiplegia | Chest CT: Mild pneumothorax; Gas in coronary arteries; Brain CT: Minimal gas in cerebral arteries | High-flow oxygen therapy; Fluid resuscitation | Recovered |
| 2013^[28]^ | S. Perinel Ragey et al. | Male | 70 | Dyspnea | Lung cancer | TBLB | Sudden consciousness disturbance; limb convulsions | Brain CT: Scattered gas in left temporal lobe | HBOT; Fluid resuscitation | Recovered |
| 2014^[29]^ | Evison et al. | Female | 84 | Weight loss | Lung cancer | TBLB | Sudden consciousness disturbance; limb convulsions | Brain CT (2h post): Gas in left frontal and parietal lobes | High-flow oxygen therapy; Fluid resuscitation | Neurological sequelae |
| 2015^[30]^ | Rocha et al. | NA | NA | Pulmonary nodule | Hepatocellular carcinoma; Hepatic fibrosis | CT-PNB | Sudden consciousness disturbance; right-sided hemiplegia | Brain CT: Gas cavity in left parietal lobe; MRI (Day5): Large bilateral infarcts | High-flow 100% oxygen; Early HBOT | Neurological sequelae |
| 2016 | Kazimirko et al. | Male | 65 | Pulmonary nodule | Lung cancer | CT-PNB | Somnolence; right-sided hemiplegia (post-anesthesia) | Brain CT: Gas in left parietal lobe | Early resuscitation; HBOT | Neurological sequelae |
| 2016^[31]^ | Akitaka Yamamoto et al. | Female | 74 | Pulmonary nodule | Breast cancer (metastatic) | CT-PNB | Cough; loss of consciousness; shock; hypoxia | Brain CT: Gas inflow in right MCA | Controlled normothermia (for neuroprotection) | Recovered |
| 2016 | HU Yan et al. | Female | 68 | Cough; Dyspnea | Lung cancer | Artificial pneumothorax for MT | Coma; unresponsiveness; pinpoint pupils | Brain CT: Scattered intraparenchymal gas | Fluid resuscitation; High-flow oxygen therapy | Neurological sequelae |
| 2017^[32]^ | Venkatkiran Kanchustambham et al. | Male | 68 | Endobronchial mass | Lung cancer | APC | Left facial droop; left-sided weakness | Brain CT: Hypodensity in right frontoparietal region (infarct); MRI: Acute-subacute cortical infarction | High-flow 100% oxygen; HBOT | Neurological sequelae |
| 2018^[33]^ | Keita Maemura et al. | Male | 77 | Pulmonary nodule | Lung cancer | TBLB | Slurred speech; left-sided hemiplegia; left-sided neglect | Brain CT: Effacement of gray-white junction in right MCA territory; Gas bubbles in MCA on mediastinal window | Normobaric oxygen (NBO₂) therapy | Neurological sequelae |
| 2018^[34]^ | Keren Fogelfeld et al. | Male | 61 | Pulmonary nodule | NA | TBLB | Seizure | CT: Evidence of hypoxic injury and cerebral edema | HBOT | Recovered |
| 2019^[35]^ | Xiao-Hua Gou et al. | Female | 55 | Dyspnea | Pleural effusion | Artificial pneumothorax for MT | Slurred speech; left limb weakness | Brain CT: Gas shadow in right cerebral vessels | HBOT | Neurological sequelae |
| 2019^[36]^ | Yong-Sheng Liu et al. | Male | 64 | Pulmonary nodule | Lung cancer | CT-PNB | Loss of consciousness; convulsions | Brain CT: Branching/linear gas distribution in right MCA territory | HBOT; Antiplatelet therapy; Dehydration therapy | Died |
| 2019^[37]^ | L. Hellinger et al. | Male | 74 | Pulmonary nodule | Lung cancer | CT-PNB | Cardiovascular collapse | CT: Gas in left ventricle, aorta, spinal canal, and brain parenchyma | Epinephrine; Chest compressions; HBOT | Died |
| 2019^[38]^ | Hikaru Toyota et al. | Female | 88 | Acquired drug resistance (to Gefitinib) | Lung cancer | TBLB | Bradycardia; ST elevation; unresponsiveness; conjugate gaze deviation; left-sided hemiplegia | Brain CT: Several round lucencies in left hemisphere | HBOT | Recovered |
| 2019^[39]^ | Francesca Piccoli et al. | Female | 36 | Pulmonary nodule (on FDG-PET) | Localized secondary Hodgkin lymphoma | CT-PNB | Hemoptysis; tonic-clonic seizure | Brain CT: Mild cerebral edema; Bilateral intracranial gas, mainly in right frontal and parietal lobes | High-flow oxygen; External ventricular drain (for ICP control); Medically induced coma (48h) | Recovered |
| 2020^[40]^ | Marvin Meßemer et al. | Male | 60 | Pulmonary nodule | Cerebral air embolism (secondary)。 | TBLB | Left-sided hemiplegia (predominant) | Brain CT: Two small gas bubbles in periventricular and frontal cortex of right hemisphere | Systemic thrombolysis | Neurological sequelae |
| 2020^[41]^ | M. Agossou et al. | Female | 80 | Pulmonary nodule | Malignancy with metastasis | TBLB | Confusion; pinpoint pupils | Brain CT: Diffuse cerebral air embolism | HBOT | Died |
| 2020^[42]^ | Katrien Van Den Plas et al. | NA | 78 | Pulmonary nodule | Benign lymph node | TBLB | Unresponsiveness; bilateral Babinski sign positive | Brain CT: Small, round, serpiginous lucencies in left occipital region | HBOT | Neurological sequelae |
| 2020^[43]^ | Hyung‐Joo Oh et al. | Female | 80 | Pulmonary nodule | Lung cancer | CT-PNB | Altered mental status; cyanosis | Brain CTA: Major vessels patent; No evidence of thrombotic or air embolic infarction | Intravenous fluid resuscitation; Endotracheal intubation; 100% oxygen ventilation | Recovered |
| 2021^[44]^ | Vladimir Herout et al. | Male | 70 | Pulmonary nodule | NA | TBLB | Left-sided hemiplegia | Brain CT: Gas in venous system of right hemisphere | HBOT | Died |
| 2021^[45]^ | Dissanayake et al. | Male | 75 | Intraplecular catheter (IPC) with foul-smelling drainage | Pleural effusion | IPC exchange | Decreased consciousness; seizure-like activity | Brain CT: Multiple serpiginous gas tracts along cerebral fissures | High-flow oxygen therapy; Head-down positioning | Died |
| 2021^[46]^ | Yu-Ping He et al. | Male | 56 | Lung cancer (known diagnosis) | Lung cancer | Bronchoscopy-guided thermal ablation | Unresponsiveness; hypoxia; bradycardia; quadriplegia | Brain CT: Several low-density plaques (gas) in right frontal lobe arteries | HBOT | Recovered |
| 2022^[47]^ | Sokol Bilali et al. | NA | 85 | Persistent cough; Sputum | Pulmonary infection | Diagnostic fiberoptic bronchoscopy | Speech difficulty; somnolence; convulsions | Brain CT: Multiple round lucencies at gray-white junction | Intravenous anticonvulsants; Endotracheal intubation | Died |
| 2022^[48]^ | Ishihara et al. | Male | 40 | Fever; Chest pain; Dyspnea | Empyema | Pleural Lavage | Loss of consciousness | Brain CT: Cerebral air embolism | HBOT | Recovered |
| 2022^[49]^ | Huajie Xing et al. | Male | 49 | Pulmonary nodule | NA | CT-PNB | Left-sided limb paralysis | CT: Small gas bubbles in ascending aorta and branch V2b | HBOT; Intensive rehabilitation | Recovered |
| 2022^[50]^ | Mafalda Silva et al. | Male | 69 | Pulmonary nodule | NA | CT-PNB | Loss of consciousness; left limb paralysis | Brain imaging: Cerebral air embolism | HBOT | Recovered |
| 2023^[51]^ | Dazhi Guo et al. | Female | 65 | Pulmonary nodule | NA | CT-PNB | Loss of consciousness; convulsions; arrhythmia | Brain CT: Branching/linear gas in right temporal, parietal, occipital lobes and left frontal, parietal lobes | HBOT; Decompressive craniectomy | Neurological sequelae |
| 2023^[52]^ | André Santos et al. | Male | 54 | Pulmonary nodule | Benign pulmonary nodule | TNB | Loss of consciousness; followed by dysarthria; right arm weakness | Brain CT: Small amount of gas in left parieto-occipital subarachnoid space | High-flow oxygen therapy | Recovered |
| 2024^[53]^ | Federica Ricciardella et al. | Male | 75 | Pulmonary nodule | Lung cancer | CT-PNB | Hemoptysis; loss of consciousness | Brain CT: Gas microbubbles in subarachnoid space over bilateral hemispheric convexities | NA | NA |
| Note: Data are presented as reported in the cited references.  Abbreviations: APC, argon plasma coagulation; BOOP, bronchiolitis obliterans with organizing pneumonia; CPR, cardiopulmonary resuscitation; CT-PNB, CT-guided percutaneous needle biopsy; GCS, Glasgow Coma Scale; HBOT, hyperbaric oxygen therapy; IPC, indwelling pleural catheter; MT, medical thoracoscopy; NBO₂, normobaric oxygen therapy; TBLB, transbronchial lung biopsy; TBNA, transbronchial needle aspiration; TNB, transthoracic needle biopsy; ACA, anterior cerebral artery; MCA, middle cerebral artery; NA, not available. | | | | | | | | | | |

REFERENCES:

1. Wang LZ, et al. Acute cerebral infarction complicating percutaneous lung puncture: a case report. Chin J Tubere Respir Dis 2007;30:475. doi: 10.3760/cma.j.issn.1001-0939.2007.06.020 (in Chinese).
2. Ke CX, et al. Observation and nursing of complications in medical thoracoscopy. J Nurs Sci 2008;23:46-47 (in Chinese).
3. Yu XW, et al. Nursing discussion on complications of thoracoscopic interventional surgery. Chin J Mod Nurs 2009;15:2672-2673 (in Chinese).
4. Sun L, Zhang J, Wang J, et al. Complications and prevention strategies in medical thoracoscopy. Chin J Respir Crit Care Med 2019;18:519-522. doi: 10.7507/1671-6205.201805001 (in Chinese).
5. Zhou XY, et al. Diagnostic value of medical thoracoscopy for tuberculous pleural effusion. Chin J Antituberc 2015;37:948-952 (in Chinese).
6. Song XH, et al. Rescue of a case of air embolism secondary to CT-guided percutaneous lung biopsy. J Nurs 2016;31:89-91. doi: 10.3870/j.issn.1001-4152.2016.18.089 (in Chinese).
7. Wang ZH, et al. A case of cortical laminar necrosis caused by air embolism complicating percutaneous lung biopsy. Chin J Neurol 2020;53:213-214. doi: 10.3760/cma.j.issn.1006-7876.2020.03.012 (in Chinese).
8. Yang ZH, et al. Successful hyperbaric oxygen therapy for two special cerebrovascular diseases. Chin J Naut Med Hyperb Med 2020;27:116-117 (in Chinese).
9. Kodama F, Ogawa T, Hashimoto M, Tanabe Y, Suto Y, Kato T. Fatal air embolism as a complication of CT-guided needle biopsy of the lung. J Comput Assist Tomogr 1999;23:949-951. doi: 10.1097/00004728-199911000-00022.
10. Ohashi S, Endoh H, Honda T, Komura N, Satoh K. Cerebral air embolism complicating percutaneous thin-needle biopsy of the lung: complete neurological recovery after hyperbaric oxygen therapy. J Anesth 2001;15:233-236. doi: 10.1007/s005400170008.
11. Shetty PG, Fatterpekar GM, Manohar S, Sujit V, Varsha J, Zarir U. Fatal cerebral air embolism as a complication of transbronchoscopic lung biopsy: a case report. Australas Radiol 2001;45:215-217. doi: 10.1046/j.1440-1673.2001.00905.x.
12. Ferretti G, Lavagne P, Delafosse B. Embolie gazeuse compliquant une ponction transthoracique à l'aiguille à couperet. J Radiol 2004;85:1067-1069. doi: 10.1016/S0221-0363(04)97721-9.
13. Timpert K, et al. Massive zerebrale luftembolie nach computertomographie-gesteuerter lungenbiopsie. RöFo 2006;178:441-443. doi: 10.1055/s-2006-926624.
14. Hiraki T, Fujiwara H, Sakurai J, Iguchi T, Gobara H, Tajiri N, et al. Nonfatal systemic air embolism complicating percutaneous CT-guided transthoracic needle biopsy. Chest 2007;132:684-690. doi: 10.1378/chest.06-3030.
15. Hirasawa S, Hirasawa H, Taketomi-Takahashi A, Morita H, Tsushima Y, Amanuma M, et al. Air embolism detected during computed tomography fluoroscopically guided transthoracic needle biopsy. Cardiovasc Intervent Radiol 2008;31:219-221. doi: 10.1007/s00270-006-0260-z.
16. Kau T, Rabitsch E, Celedin S, Habernig SM, Weber JR, Hausegger KA. When coughing can cause stroke—a case-based update on cerebral air embolism complicating biopsy of the lung. Cardiovasc Intervent Radiol 2008;31:848-853. doi: 10.1007/s00270-008-9339-z.
17. Tomabechi M, Kato K, Sone M, Ehara S, Sekimura K, Kizawa T, et al. Cerebral air embolism treated with hyperbaric oxygen therapy following percutaneous transthoracic computed tomography-guided needle biopsy of the lung. Radiat Med 2008;26:379-383. doi: 10.1007/s11604-008-0242-y.
18. Hsi DH, et al. Simultaneous coronary and cerebral air embolism after CT-guided core needle biopsy of the lung. Tex Heart Inst J 2008;35:472-474.
19. Osseiran K, Barchfeld T, Dellweg D, Haidl P. Zerebrale arterielle gasembolie als komplikation während einer therapeutischen endobronchialen anwendung der argon-plasma-koagulation. Pneumologie 2008;62:353-354. doi: 10.1055/s-2008-1038133.
20. Um SJ, Lee SK, Yang DK, Son C, Kim KN, Lee KN, et al. Four cases of a cerebral air embolism complicating a percutaneous transthoracic needle biopsy. Korean J Radiol 2009;10:81-84. doi: 10.3348/kjr.2009.10.1.81.
21. Azzola A, Von Garnier C, Chhajed PN, Schirp U, Tamm M. Fatal cerebral air embolism following uneventful flexible bronchoscopy. Respiration 2010;80:569-572. doi: 10.1159/000321849.
22. Bou-Assaly W. Systemic air embolism after transthoracic lung biopsy: a case report and review of literature. World J Radiol 2010;2:193-196. doi: 10.4329/wjr.v2.i5.193.
23. Thomas R, Thangakunam B, Cherian RA, Gupta R, Christopher DJ. Cerebral air embolism complicating CT-guided trans-thoracic needle biopsy of the lung. Clin Respir J 2011;5:e1-3. doi: 10.1111/j.1752-699X.2010.00229.x.
24. Shaw Y, Yoneda KY, Chan AL. Cerebral gas embolism from bronchoscopic argon plasma coagulation: a case report. Respiration 2012;83:267-270. doi: 10.1159/000328939.
25. Shi L, Zhang R, Wang Z, Zhou P. Delayed cerebral air embolism complicating percutaneous needle biopsy of the lung. Am J Med Sci 2013;345:501-503. doi: 10.1097/MAJ.0b013e31827bbe23.
26. Suzuki K, Ueda M, Muraga K, Abe A, Suda S, Okubo S, et al. An unusual cerebral air embolism developing within the posterior circulation territory after a needle lung biopsy. Intern Med 2013;52:115-117. doi: 10.2169/internalmedicine.52.8760.
27. Smit DR, Kleijn SA, de Voogt WG. Coronary and cerebral air embolism: a rare complication of computed tomography-guided transthoracic lung biopsy. Neth Heart J 2013;21:464-466. doi: 10.1007/s12471-013-0411-1.
28. Perinel Ragey S, Garnier P, Vergnon JM. Complete resolution of cerebral air embolism secondary to a transbronchial needle aspiration. Respiration 2013;86:504-507. doi: 10.1159/000354790.
29. Evison M, Crosbie PAJ, Bright-Thomas R, Alaloul M, Booton R. Cerebral air embolism following transbronchial lung biopsy during flexible bronchoscopy. Respir Med Case Rep 2014;12:39-40. doi: 10.1016/j.rmcr.2013.10.005.
30. Rocha RD, Azevedo AA, Falsarella PM, Rahal A, Garcia RG. Cerebral air embolism during CT-guided lung biopsy. Thorax 2015;70:1099-1100. doi: 10.1136/thoraxjnl-2015-207205.
31. Yamamoto A, Suzuki K, Iwashita Y, Yokoyama K, Omori Y, Suzuki H, et al. Controlled normothermia for a cerebral air embolism complicating computed tomography-guided transthoracic needle biopsy of the lung. Acute Med Surg 2016;3:411-414. doi: 10.1002/ams2.211.
32. Reddy M, Patolia S. Cerebral air embolism as possible cause of stroke during therapeutic endobronchial application of argon plasma coagulation. Cureus 2017;9:e1255. doi: 10.7759/cureus.1255.
33. Maemura K, Kage H, Isago H, Takeshima H, Makita K, Amano Y, et al. Cerebral arterial air embolism after diagnostic flexible fiberoptic bronchoscopy: a case report and review of the literature. Case Rep Pulmonol 2018;2018:7108215. doi: 10.1155/2018/7108215.
34. Fogelfeld K, Rana RK, Soo Hoo GW. Cerebral artery gas embolism following navigational bronchoscopy. J Intensive Care Med 2018;33:536-540. doi: 10.1177/0885066618766838.
35. Gou XH, Yang W, Zhang YL, Li Y, Wu X. A case of massive cerebral arterial air embolism induced by artificial pneumothorax and its analysis. Med Princ Pract 2019;28:297-300. doi: 10.1159/000496342.
36. Liu YS, An XB, Li K, Liu YJ, Wang F. Cerebral arterial air embolism after computed tomography-guided hook-wire localization of a pulmonary nodule: a case report. Medicine (Baltimore) 2019;98:e15437. doi: 10.1097/MD.0000000000015437.
37. Hellinger L, Keppler AM, Schoeppenthau H, Perras J, Bender R. Hyperbaric oxygen therapy for iatrogenic arterial gas embolism after CT-guided lung biopsy: a case report. Anaesthesist 2019;68:456-460. doi: 10.1007/s00101-019-0618-7.
38. Toyota H, Kobayashi K, Matsukura S, Kawamura Y, Kanda T, Arai H, et al. A case of cerebral air embolism during diagnostic flexible bronchoscopy. J Bronchology Interv Pulmonol 2019;26:e58-e60. doi: 10.1097/LBR.0000000000000608.
39. Piccoli F, Lanza E, Lutman RF. Cerebral air embolism after CT-guided lung biopsy: a case of early diagnosis and successful treatment. Arch Bronconeumol (Engl Ed) 2019;55:599-600. doi: 10.1016/j.arbres.2019.04.003.
40. Meßemer M, Waibel S, Weininger M. 60-jähriger Patient mit Hemiparese links nach Bronchoskopie. Dtsch Med Wochenschr 2020;145:655-656. doi: 10.1055/a-1113-1121.
41. Agossou M, Holtea-Souty D, Fore M, Vuillemin C, Debieuvre D. Embolie gazeuse cérébrale : une complication rare de la fibroscopie bronchique souple. Rev Mal Respir 2020;37:662-665. doi: 10.1016/j.rmr.2020.07.003.
42. Van Den Plas K, Van Den Bergh V, Van Grimberge F, Legius B, Decoster L. Cerebral arterial air embolism after endobronchial ultrasound-guided transbronchial needle aspiration. J Bronchology Interv Pulmonol 2020;27:e62-e64. doi: 10.1097/LBR.0000000000000676.
43. Oh HJ, Jeong WG, Lim Y, Koh SJ, Lee SM, Kim MS, et al. Potentially fatal complications of systemic air embolism after computed tomography-guided transthoracic needle biopsy in lung cancer harboring epithelial growth factor receptor mutation: a case report. Thorac Cancer 2020;11:3401-3406. doi: 10.1111/1759-7714.13686.
44. Herout V, Brat K, Richter S, Cierny IC Jr. Cerebral air embolism complicating transbronchial lung biopsy: a case report. World J Clin Cases 2021;9:9911-9916. doi: 10.12998/wjcc.v9.i32.9911.
45. Jayawardena DMC, Panchal RK, Agrawal S, Das I. Cerebral air embolism after indwelling pleural catheter insertion in a chronic hydropneumothorax secondary to epithelioid mesothelioma. BMJ Case Rep 2021;14:e244006. doi: 10.1136/bcr-2021-244006.
46. He YP, Liu YL, Gao XL, Wang LH. Cerebral arterial air embolism after endobronchial electrocautery: a case report and review of the literature. BMC Pulm Med 2021;21:222. doi: 10.1186/s12890-021-01580-w.
47. Bilali S, Bilali V, Saraci B, Zekja I, Nina H. Fatal air embolism: a grave complication during diagnostic flexible bronchoscopy. Clin Case Rep 2022;10:e05287. doi: 10.1002/ccr3.5287.
48. Ishihara T, Sato S, Manabe S, Ozawa H. Complications of cerebral air embolism associated with pleural lavage for empyema. BMJ Case Rep 2022;15:e249618. doi: 10.1136/bcr-2022-249618.
49. Xing H, Wang Z, Jiang Y. Case report: cerebral artery air embolism during CT-guided lung nodule resection in hybrid theater. Front Surg 2022;9:950159. doi: 10.3389/fsurg.2022.950159.
50. Silva M, Gaio-Lima C, Camacho Ó, Ribeiro J. Cerebral air embolism following transthoracic lung biopsy successfully treated with hyperbaric oxygen. Cureus 2022;14:e32933. doi: 10.7759/cureus.32933.
51. Guo D, Li D, Xue R, Lv Y, Pan S. Case report of CT-guided lung biopsy complicated by air embolism. J Emerg Med 2023;50:e1-e3. doi: 10.1016/j.jemermed.2023.01.001.
52. Santos A, Almeida C, Porto LM, Fernandes PD, Silva JP. Cerebral air embolism: a case of a rare transthoracic needle biopsy complication. Cureus 2023;15:e35203. doi: 10.7759/cureus.35203.
53. Ricciardella F, Mannetta G, Caruso V, Cocco G, Mantini C, Piccirilli E, et al. Air embolism as a rare complication of lung biopsy: a case report. Radiol Case Rep 2024;19:1547-1551. doi: 10.1016/j.radcr.2024.01.027.
